# The Impact of Verbal Encouragement and Repeating on the Measurement of Spirometry in Healthy Adult

**DOI:** 10.7759/cureus.18714

**Published:** 2021-10-12

**Authors:** Raid M AL Zhranei, Mohammad Ismail, Mohammed Allah, Mousa Alzahrani

**Affiliations:** 1 Respiratory Therapy Department, College of Applied Medical Sciences, King Saud bin Abdulaziz University for Health Sciences, Jeddah, SAU; 2 Research Office, King Abdullah International Medical Research Center, Jeddah, SAU; 3 Respiratory Therapy Administration, King Abdullah Medical City, Makkah, SAU; 4 Respiratory Therapy Department, College of Applied Medical Sciences, King Saud Bin Abdulaziz University for Health Sciences, Jeddah, SAU

**Keywords:** pulmonary function test, spirometry, repeating, verbal encouragement, vital capacity

## Abstract

Introduction

Vital Capacity (VC) is measured by spirometry. VC can differ according to weight, age, gender, race and height of the investigated person. Repetition of the same tasks has been proven to enhance adults’ performance. Encouraging on some tasks have shown to result in a significant difference.

Aim

This study aimed to examine if verbal encouragement and repetition of spirometry would make a difference in the results.

Method

This is a randomized clinical trial involving 136 healthy volunteers, randomly allocated to one of two groups: control and intervention groups. In both groups, VC was assessed as the baseline (VC1). VC was assessed again after ten days maximally (VC2). The third time of measuring VC was assessed again after ten days maximally (VC3). The verbal encouragement was provided only to the intervention group during iVC2 and iVC3, and only repeating was required from the control group in cVC2 and cVC3.

Results and Conclusion

There was no significant difference in VC after the third trial in the control group compared to the baseline measurement (p-value= 0.836). On the other hand, verbal encouragement in the third trial had a significant difference in VC compared to the baseline measurement (p-value= 0.000).

## Introduction

Spirometry is a noninvasive pulmonary function test (PFT) used to measure the volume or flow of air that moves in and out of the lungs [1]. Spirometry helps evaluate the presence or absence of lung diseases, measure the effect of disease on pulmonary function, assess preoperative risk, assess therapeutic interventions, and find the causes of lung diseases [2].

The most important values that help in spirometry measurement are forced vital capacity (FVC), forced expiratory volume in one second (FEV1), peak expiratory flow (PEF), the ratio of the forced expiratory volume in the first one second to the forced vital capacity of the lungs (FEV1/FVC) ratio, and finally the vital capacity (VC) which was the focus of this study [1]. In addition, there are two loops in spirometry, which are the flow-volume and volume-time loop.

VC is the maximum amount of air that can be exhaled when blowing out as forceful as possible after a maximal inspiration. VC in humans depends on the investigated person's weight, age, gender, race, and height [3]. For VC measurements, the patient should inhale a deep breath as much as possible and exhales it steadily as long as possible until there is no air left. The patient must use nose clips to prevent air from leaking due to the low flow [4].

In general, encouragement is effective in enhancing adults' performance. For instance, encouraging weight loss and smoking cessation has shown a significant difference. Repetition of the same task has proven to enhance adults' performance [5-7]. This research aimed to examine if verbal encouragement and repetition of spirometry would make a difference in the results.

## Materials and methods

A randomized clinical trial was carried out at King Saud bin Abdulaziz University for Health Sciences (KSAU-HS) in Jeddah campus. Healthy adult male and female students were invited to participate in this study, and the participation was voluntary. The purpose of the study was explained to the students, and they gave written informed consent. General health status and physical conditioning for all volunteers were evaluated. Volunteers who had respiratory problems, such as those with obstructive and restrictive lung diseases, hypertension or heart diseases, were excluded from the study and those who were current smokers.

**Figure 1:**
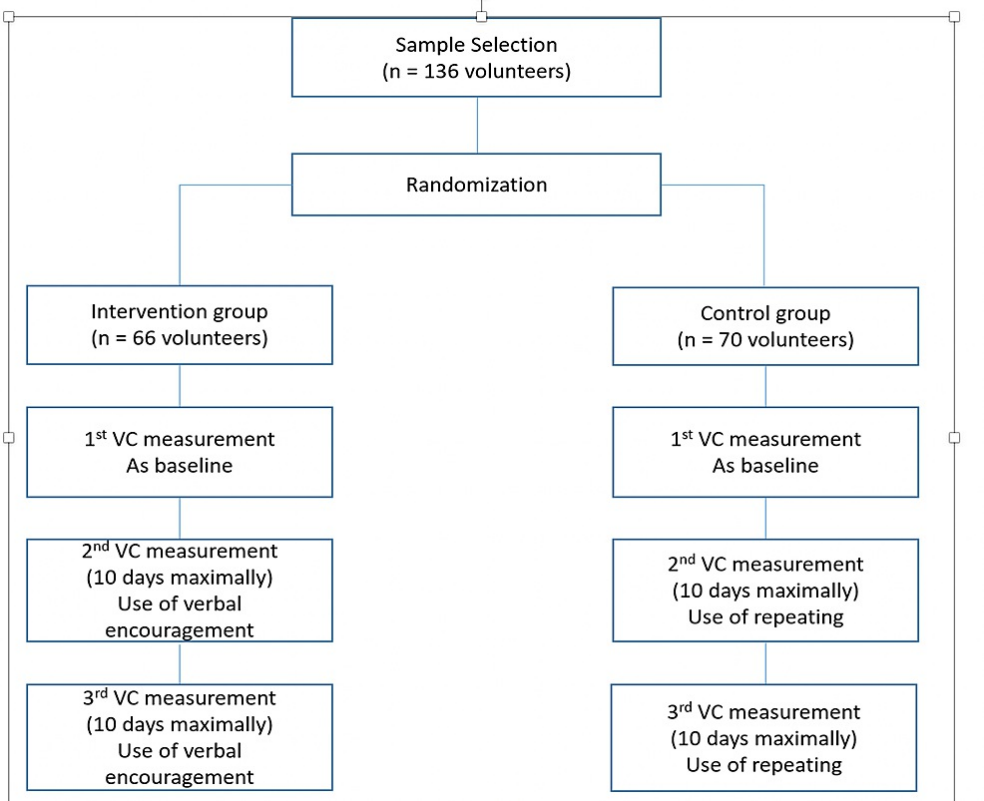
Study Protocol and Distribution of Volunteers VC- Vital Capacity

The volunteers were randomly allocated to one of two groups: the control group (cVC) or the intervention group (iVC). Randomization was performed, and it was a convent in which the odd numbers were the control group and the even numbers were the intervention group (figure 1). VC was measured three times in both groups (the control group (cVC) and the intervention group (iVC), verbal encouragement was provided only in the second and third measurement to volunteers in the intervention group (iVC), and only a repetition without encouragement was performed in the control group (cVC).

Before starting the test, instructions were given to all volun­teers on the procedures that would be performed. Following the recommendations of the European Respiratory Society (ERS), volun­teers were instructed to avoid performing a vigorous exercise within 30 minutes of testing and having a large meal within 2hours of testing. They should wear loose-fitting clothing during the tests [8].

For all participants, baseline VC (VC1) was assessed and measured with the use of the following verbal words: "Inhale deeply as much as possible and exhale as much as possible." After ten days maximally, the second assess­ment of VC (VC2) was done in both groups. However, verbal encouragement was provided only to volunteers in the intervention group. To that end, the following phrases, which is a clinical practice guideline from the Canadian Journal of Respiratory Care [9], were used to encourage the participants: "Come on! Inhale deeply! Go ahead!"; "Breathe! Breathe in, breathe in!"; "Exhale slowly. . ."; and "Out, breathe out, breathe out, breathe out completely!" After maximally ten days, the third VC (VC3) assessment was done as in (VC2). The measurements were taken and measured in (VC3) by the same technician who provided verbal encouragement (VC2).

Statistical analysis was performed using Statistical Package for the Social Sciences (SPSS) Version 20. Descriptive analysis of the data was performed. Numerical variables are presented as arithmetic median and interquartile range since they had abnormal distribution. Categorical data are shown as absolute numbers, and cate­gory frequency is presented as percentages. The Student's t-test was used to compare both groups. The level of significance was set at p < 0.05 or 5%.

The proposal of this study had been revised and approved by the ethics committee of King Abdullah International Medical Research Center (KAIMRC) with IRB number SP17/322/J.

## Results

We evaluated 136 healthy non-smokers volunteers, 76 were males (55.9%), and 60 were females (44.1%). The volunteers were randomly assigned to control and intervention groups, of whom 66 were in the intervention group, and 70 were in the control group. We focused on the characteristics of age, height, weight, and body mass index (BMI). The median age, height, weight and BMI was 21, 168, 70 and 25 with an interquartile range of 2, 13, 25 and 8, respectively (Table 1).

**Table 1:** Descriptive Statistical

Characteristics	Maiden	Interquartile Range
Age	21	2
Height	168	13
Weight	70	25
BMI	25	8

We compared the VC between two groups, and the results showed that the control group's standard deviation values were cVC1 0.90, cVC2 0.91, and cVC3 0.88. The standard deviation values for the intervention group were iVC1 0.80, iVC2 0.87, and iVC3 0.85. There was no significant difference in VC after the third trial in the control group compared to the baseline measurement (p-value= 0.836). On the other hand, verbal encouragement in the third trial resulted in a noticeable difference in VC compared to the baseline measurement (p-value= 0.000) (Table 2).

**Table 2:** Measurement of VC VC- Vital Capacity

Group	Baseline VC	VC2	VC3	p-value VC1-VC3
Control	3.7±0.90	3.6±0.9	3.7±0.88	0.836
Intervention	3.7±0.8	3.8±0.87	3.9±0.85	0.000

## Discussion

According to the results, there was an increase of 2.7% in VC from baseline (iVC1) upon the first trial of encouragement (iVC2), which was a significant difference (p-value: 0.000). A further increase (5.4%) was noticed upon the second trial of encouragement (iVC3) which was a significant difference (p-value: 0.000). This increase shows us that verbal encouragement has a significant impact on the results. On the other hand, VC decreased in the control group upon both the second and third trials, and it was not significant. The decrease could be because some samples have been tested in a short time. We found no significant difference when comparing VC in second-second and third-third trials between the intervention and control groups.

Our findings have shown that the impact of verbal encouragement is higher than repetition on the measurement of VC. There was no impact of repetition and verbal encouragement on VC regarding gender. The results have shown that with increased BMI, VC was increased. Our explanation for this was that the high BMI in our young population might reflect a more muscular body built rather than overweight, which means having stronger inspiration muscles that might help take more volume in. 

The present study results suggest that verbal encouragement should be implemented during the assessment of VC by spirometry as an effective, simple and easily applied strategy. Moreover, we found that combining the behavioural strategy with traditional practice is important to get more accurate results. 

A similar study was rolled out at the Centro Universitário Jorge Amado - UNIJORGE, Jorge Amado University Center - Salvador, Brazil [10]. Their results showed an increase in VC in the intervention group, similar to our results. Contrary to our results, their results showed an increase in VC of the control group, which might be because of getting a larger sample size that included a similar number of gender as opposed to them.

Verbal encouragement is the most effective way to get more accurate results compared to repetition. Moreover, it is a simple method in clinical practice to save time and effort. So, we recommend applying verbal encouragement during spirometry to get better results. Furthermore, we suggest that those interested in researching VC apply verbal encouragement during spirometry. In addition, taking a larger, random sample might reveal better results.

Our strength point of this study is one of the very few studies that investigated this idea.

There are two significant limitations in this study that could be addressed: a lack of prior research studies on the topic. Second, the majority of the sample in size in this study were university students.

## Conclusions

Verbal encouragement is more effective than repetition in the results of spirometry. Moreover, we found that combining the behavioural strategy with traditional practice is important to get more accurate results. We suggest that those interested in researching VC apply verbal encouragement during spirometry. In addition, taking a larger, random sample might reveal better results.
